# Relationship of Morning Cortisol to Circadian Phase and Rising Time in Young Adults with Delayed Sleep Times

**DOI:** 10.1155/2012/749460

**Published:** 2012-10-22

**Authors:** Mark S. Rea, Mariana G. Figueiro, Katherine M. Sharkey, Mary A. Carskadon

**Affiliations:** ^1^Lighting Research Center, Rensselaer Polytechnic Institute, 21 Union Street, Troy, NY 12180-3352, USA; ^2^Department of Psychiatry & Human Behavior, Alpert Medical School of Brown University, Box G-EPB, Providence, RI 02918, USA; ^3^Department of Medicine, Division of Pulmonary, Critical Care and Sleep Medicine, Alpert Medical School of Brown University, Box G-RIH, Providence, RI 02918, USA

## Abstract

The present study was aimed at further elucidating the relationship between circadian phase, rising time, and the morning cortisol awakening response (CAR). The results presented here are a secondary analysis of experimental data obtained from a study of advanced sleep-wake schedules and light exposures on circadian phase advances measured by dim-light melatonin onset (DLMO). The present results demonstrate that morning CAR is strongly related to rising time and more weakly related to DLMO phase.

## 1. Introduction

Melatonin synthesis by the pineal gland represents one circadian rhythm controlled by the suprachiasmatic nuclei (SCN) of the hypothalamus. The light-dark pattern incident on the retina sets the timing of the SCN, which in turn restricts the timing of melatonin synthesis to the dark phase of the 24-hour cycle (i.e., during subjective night). Across mammalian species, melatonin concentrations are highest at night irrespective of the photic niche of the animal—nocturnal, crepuscular, or diurnal. With the exception of retinal light exposure, neither environmental (e.g., exercise, diet) nor endogenous (e.g., sleep) stimuli affect melatonin synthesis.

Corticosteroid production by the adrenal gland also exhibits a robust circadian pattern in mammals. In contrast to melatonin production, however, the timing of corticosteroid synthesis is closely related to the photic niche of the species, with peak levels occurring at the daily transition from quiescence to activity. Thus, peak levels of corticosteroids in diurnal mammals occur in the morning, whereas peak levels occur in the evening for nocturnal mammals. The daily pattern of corticosteroid production is influenced by several interacting systems, only one of which is the master clock in the SCN. Recently, Clow and colleagues [1] proposed a three-part control mechanism for the cortisol awakening response (CAR) in humans. At least two of these three mechanisms are explicitly under the influence of the SCN: a preawakening reduced sensitivity to adrenocorticotropic hormone (ACTH) and a postawakening enhanced sensitivity to ACTH. The third mechanism, influenced by the SCN, is a light-sensitive sympathetic pathway to the adrenal that is independent of the pituitary [2, 3]. Although regulation of the adrenal is complex, with the multiple control mechanisms that can be differentially important to corticosteroid production, the SCN plays an important role in anticipatory corticosteroid production [4].

Under natural conditions, one might expect a close correspondence between circadian time, as measured by a reliable marker like dim light melatonin onset (DLMO) phase, and the awakening time. In fact, Burgess and Eastman [5, 6] as well as Crowley and colleagues [7] have shown that wake times and DLMO are correlated when participants are on an unrestricted schedule. On the other hand, Wilhelm and colleagues [8] have shown that CAR is relatively unaffected by circadian time. Rather, they concluded that CAR is determined mainly by the act of awakening from sleep. Nevertheless, these authors readily conceded that CAR has some relationship to circadian time because of the known neural (albeit indirect) connections between the SCN and the adrenal [3] and because empirical evidence demonstrates that the amplitude of the CAR varies with circadian time [1].

The purpose of the present analysis was to examine the association of circadian phase (measured from salivary DLMO time) with the time of peak cortisol in the morning during a two-week study of late-sleeping young adults. This report is a secondary analysis of a parent study [9] that tested the effects of an advanced sleep-wake schedule and a morning light exposure intervention on circadian phase advances. The protocol allowed us to examine CAR in young adults before and after an advance in the sleep-wake schedule and attempt to dissociate the influences of sleep timing and circadian phase on CAR.

## 2. Methods

Twenty-five participants (ages 18–30; mean ± standard deviation (SD) was 21.8 ± 3.0 years; 13 females) who reported a misalignment between their self-reported sleep-wake times and their obligatory morning work or study schedules at least once per week completed the study. All participants were considered to be otherwise healthy and without any medical concern or drug use; specific details regarding race, gender, psychological disorders, affect (positive and negative), drug use, and bed times are provided in Sharkey et al. [9].

After a baseline week on their usual schedules, participants were placed on individualized sleep-wake schedules for another week. Participants were instructed to maintain a strict phase-advanced, 7.5-hour sleep duration schedule for the second week of the experiment. They were required to be in bed, in the dark, and to try to sleep during the scheduled sleep times. The advanced schedules were designed to enable participants to meet their obligatory work or school commitments. Napping was prohibited throughout both study weeks. Wrist actigraphs (Ambulatory Monitoring, Inc., Ardsley, NY, USA) were worn continuously to ensure protocol compliance.

Participants were randomly assigned to receive morning “blue-light” or “dim-light” for the second week. Both groups sat with a commercially available light-treatment device containing short-wavelength (blue) light-emitting diodes (Apollo P2 GoLite, Philips Respironics, Pittsburgh, PA, USA). The blue-light group (*n* = 12; 6 females) faced the light-treatment device, receiving approximately 225 lux of short-wavelength light (*λ*_max⁡_ ≈ 470 nm; full width half maximum ≈20 nm) at the corneas for one hour after rising in the morning, while the dim-light group (*n* = 13; 7 females) received less than 1 lux of the same blue light because the light-treatment device was positioned orthogonal to their line of gaze during the light exposure. Compliance with morning light exposure was verified with the Daysimeter [10]. The Daysimeter is a headset device that is used to continuously measure circadian light, or CL_A_, exposures.

At the end of the baseline week and again following the postintervention week (Friday evenings), participants came to the laboratory for an overnight session that included evening saliva collection for circadian phase assessment (DLMO) and morning saliva collection for determining cortisol levels. The timing of evening saliva collection was determined by each participant's predicted baseline DLMO phase, calculated from self-reported sleep schedules using the algorithm developed by Burgess and Eastman [5]. Participants used salivettes (Sarstedt, Nümbrecht, Germany) to give 1 mL saliva samples for melatonin and cortisol assays. Samples for melatonin assay were taken every 30 minutes from 3.5 hours before the predicted DLMO to 2 hours after predicted baseline DLMO. Samples for cortisol assay were obtained immediately upon awakening and every subsequent hour for 4 hours. All saliva samples were frozen at −20°C and were later radioimmunoassayed for evening melatonin and morning cortisol (Alpco, Salem, NH, USA). All samples from an individual participant were assayed with the same kit. This study was approved by the Institutional Review Board (IRB) at Rhode Island Hospital and by the IRB at Rensselaer Polytechnic Institute; participants were paid for their participation in the study [11]. Additional details of the study methods and results can be found in Sharkey et al. [9].

### 2.1. Data Analyses

The area under the curve (AUC) was calculated based on the method described by Pruessner et al. [12] whereby samples collected at 0 (upon awakening), 60, 120, 180, and 240 minutes postawakening are all included. DLMO phase and AUC were subjected to mixed design (2 groups, dim-light and blue-light), repeated measures (2 weeks) Analyses of Variance (ANOVAs) (PASW Statistics, 18.0). Post hoc, two-tailed Student's *t*-tests were performed to test the main effects and interactions between the variables. Regression analyses were performed to evaluate the associations among peak morning cortisol time, circadian phase (DLMO time), and rising time. Goodness of fit was evaluated using the *R* ^2^ values and an *F*-test was used to check the statistical significance of the overall fit (PASW Statistics, 18.0).

## 3. Results

### 3.1. General

Home monitoring confirmed that all participants complied with the prescribed protocol; those in the dim-light and blue-light groups followed instructions with regard to usage of the light-treatment device. Based upon visual inspection of the actigraphy data, sleep logs, telephone call-ins to the laboratory, and self-reports, all participants went to bed and arose at the prescribed times and there was no evidence that participants awoke during the prescribed sleep opportunity period.

### 3.2. Circadian Phase Shifts (DLMO) and Light Exposures

As previously reported [9], the time of DLMO showed a significant main effect of weeks (*F*_1,23_ = 61.4, *P* < 0.001), but the main effect of groups and the groups-by-weeks interaction were not statistically significant (*F*_1,23_ = 1.27, n.s. and *F*_1,23_ = 0.05, n.s., resp.). On average, both groups of subjects exhibited a 1.4 ± 0.9 h phase advance of DLMO. The mean ± DLMO times for all subjects were 23 : 24 ± 1 : 16 h after week 1 and 21 : 59 ± 1 : 03 h after week 2. The morning blue-light treatment did not differentially affect the time of DLMO during week 2 with respect to a dim-light exposure at the same time. We examined daily light exposures from the Daysimeter for the two groups and found that they were not significantly different between groups. Mixed design ANOVAs using the total (log) circadian light (CL_A_) exposures (as detailed in [9]) showed no significant main effect of groups (*F*_1,22_ = 0.059, n.s.), of weeks (*F*_1,22_ = 0.037, n.s.), nor a significant groups-by-weeks interaction (*F*_1,22_ = 0.134, n.s.).

### 3.3. Cortisol

#### 3.3.1. AUC

**Figure 1:**
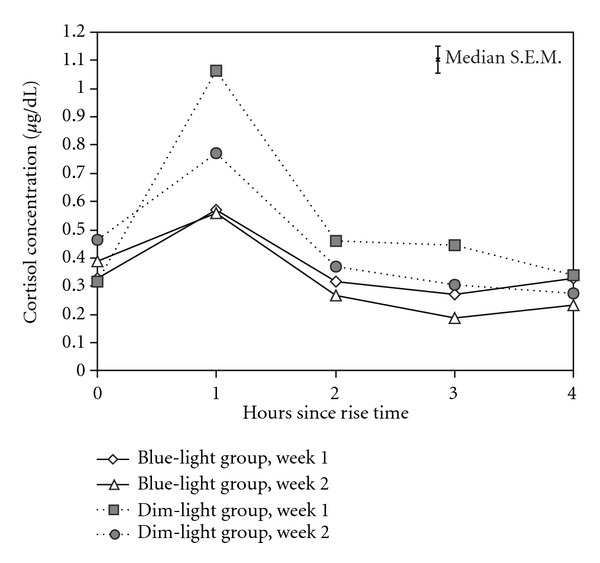
Mean morning cortisol concentrations collected at the end of week 1 and of week 2 for both groups collected at rise time (zero hours since rise time) and for 4 consecutive hours after rising. Open symbols refer to the blue-light group (diamonds = week 1 and triangles = week 2) and closed symbols refer to the dim-light group (squares = week 1 and circles = week 2). A median value for the standard error of the means (S.E.M.) is shown on the top right quarter of the graph.

Figure 1 shows the mean morning cortisol concentrations obtained from both groups during both weeks. Using AUC as the dependent measure, we found a significant main effect of weeks (*F*_1,23_ = 12.6, *P* = 0.002) and a significant main effect of groups (*F*_1,23_ = 15.9, *P* = 0.001), but the groups-by-weeks interaction was not statistically significant (*F*_1,23_ = 3.3, n.s.). During week 1, the mean ± SD morning cortisol AUC was 2.3 ± 0.6 *μ*g/dL for the dim-light group and 1.5 ± 0.5 *μ*g/dL for the blue-light group. During week 2, the mean ± SD morning cortisol AUC was 1.8 ± 0.5 *μ*g/dL for the dim-light group and 1.3 ± 0.2 *μ*g/dL for the blue-light group.

#### 3.3.2. Associations among Peak Cortisol Time, Rising Time, and DLMO Phase

**Figure 2:**
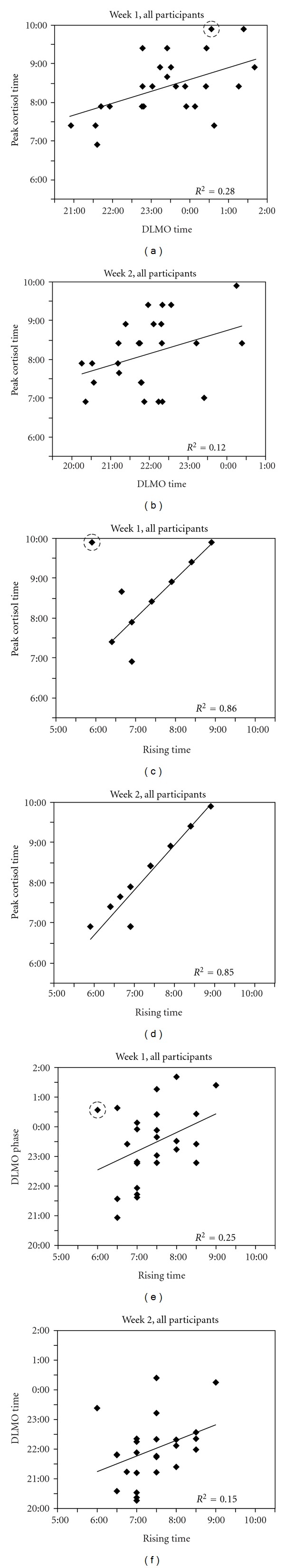
Peak cortisol time plotted as a function of circadian phase, DLMO (top row), and as a function of rising time (middle row), as well as DLMO plotted as a function of rising time (bottom row) on week 1 (left column) and on week 2 (right column). The data from the baseline week for one subject, the circled points in the left column of plots, were omitted from the regression analyses; see text.

Linear regression analyses were used to test the strengths of associations among the measured times of peak cortisol concentration, rising, and DLMO. These associations are illustrated by the six plots in Figure 2 for both study weeks. The maximum cortisol concentrations were obtained from participants one hour after rising 86% of the time. However, the peak cortisol concentration times obtained from one subject during the first baseline week was at 10:00 h, four hours after rising. It seems unlikely that morning peak cortisol levels would have occurred four hours after rising, particularly since the second highest cortisol concentration was one hour after rising for this subject, like the peak for nearly all of the other subjects during both study weeks. Thus, the circled values in Figure 2 from this one subject during the baseline week were omitted from the regression analyses.

DLMO phase was significantly associated with peak cortisol concentration time on week 1 (*R* ^2^ = 0.28; *F*_1,22_ = 8.76; *P* = 0.007) but not on week 2 (*R* ^2^ = 0.12; *F*_1,23_ = 3.14; n.s.) as shown in the top row of plots in Figure 2. Peak cortisol concentration time was, however, strongly associated with rising time both on week 1 (*R* ^2^ = 0.86; *F*_1,22_ = 140; *P* < 0.0001) and on week 2 (*R* ^2^ = 0.85; *F*_1,23_ = 133; *P* < 0.0001) as shown in the second row of plots in Figure 2. The associations between rising time and DLMO phase for both weeks are shown in the bottom row of plots. On week 1, the association was statistically significant (*R* ^2^ = 0.25; *F*_1,22_ = 7.18; *P* = 0.01), while it was not on the week two (*R* ^2^ = 0.15; *F*_1,23_ = 4.11; n.s.).

## 4. Discussion

As implied by the findings from Wilhelm and colleagues [8], the present study shows that the timing of morning peak cortisol secretion was more strongly associated with awakening than with circadian phase position, as measured by DLMO time.

Burgess and Eastman [5] as well as Crowley et al. [7] showed a much higher correlation between rising time and DLMO phase than was shown here. Both groups found a strong correlation between rising time and DLMO phase for subjects on unrestricted sleep schedules. Importantly, however, both laboratories also found a lower correlation between rising time and DLMO phase for subjects on strict sleep schedules. This weak relationship between rising time and DLMO phase was also shown during the second week of the present study, consistent with these earlier findings [5–7]. Since the relationship between DLMO phase and rising time is also relatively low, although statistically significant, during the first week of the present study, it is perhaps reasonable to infer that the subjects in the present study were not necessarily on a completely unrestricted sleep schedule. It will be recalled that these subjects were selected for participation in the study because their work and school schedules were sometimes at odds with their natural rising time. On the other hand, because of the sampling constraints for the study, intersubject sleep schedule variability may have been lower, thus reducing the correlation with DLMO phase due to restricted range.

The significant change in cortisol AUC between weeks may reflect the advanced circadian phase. In other words, the advance in circadian phase during the second week could have systematically shifted the morning downward slope of cortisol concentration to an earlier time relative to the fixed waking schedule, resulting in lower cortisol AUC between weeks. Alternatively, habituation to the experiment and/or placing the subjects on a regular sleep-wake pattern during the second week may have reduced their “stress” levels with consequent lower cortisol AUC between weeks. On the other hand, self-assessment questionnaires showed no evidence that subjects in either group reported a reduction in stress from already low values in the second week. An explanation for significant differences in cortisol AUC between groups and study weeks remains unresolved.

From a clinical perspective, notwithstanding the unresolved differences between morning cortisol levels in the two groups, these data and those by Burgess and Eastman [5] and by Crowley et al. [7] suggest future research to determine whether it is important to coordinate personal light exposures with scheduled sleep. Circadian phase is determined by the daily retinal light exposure, but the scheduled time of waking can be unrelated to daily light exposures. In general, and indeed as shown by Sharkey and colleagues [9], changing sleep schedule will produce concomitant changes in the patterns of daily retinal light exposures. Broadly then, adjusting bedtime and wake time will, albeit indirectly, affect circadian phase. Once the first-order magnitude change in light patterns due to scheduling has adjusted circadian phase (e.g., changing time zones), idiosyncratic behavior, with opportunities for differences in personal retinal light exposures from both natural and electric lighting during waking hours, will undoubtedly produce second-order changes to circadian phase. Viewing a sunrise one morning (but not the next) or reading in bed one evening (but not the next) can change a person's circadian phase from one day to the next. Rising according to a fixed schedule will not, therefore, be perfectly correlated with these second-order, idiosyncratic changes in circadian phase. Consequently, the correlation between circadian phase (DLMO) and morning peak cortisol (CAR) would be expected to be, and indeed was in the present study, lower during the second week with fixed sleep schedules than during the first week with more “natural” sleep schedules. Whether this second-order misalignment between circadian phase and morning cortisol peak has clinical significance is unknown. As reported by Sharkey and colleagues [9], there were no significant changes in the psychological well-being of subjects from the first week to the second week despite the drop in the correlation between DLMO and CAR. It remains a subject for future study to determine *if* misalignment between circadian phase and fixed rising times has clinical significance and, if it does, *how* that misalignment might be effectively and practically resolved with controlled light exposures.

In summary, the present study demonstrates that rising time and peak cortisol concentration time, usually CAR, are closely related, whereas rising time and DLMO phase are less well-correlated, especially for individuals on restricted sleep schedules. Thus, neither CAR nor rising time should be considered unambiguous markers of circadian phase, although rise times can be used to estimate a window within which sampling may detect DLMO phase. Thus, the results of the present study are consistent with the findings from Wilhelm et al. [8], as well as those from Burgess and Eastman [5] and Crowley et al. [7]. Moreover, the recommendations put forward from the latter groups for estimating DLMO phase using rising time are applicable for individuals with either unrestricted or restricted sleep schedules, recognizing the larger uncertainty in those estimates for individuals in the latter group. Finally, it would appear important for future clinical research to determine if it is necessary to be concerned with the misalignment of circadian phase and peak morning cortisol and, if it is important, how the light-dependent circadian phase can be controlled so that it can be coordinated with scheduled waking.

## References

[1] Clow A, Hucklebridge F, Stalder T, Evans P, Thorn L (2010). The cortisol awakening response: more than a measure of HPA axis function. *Neuroscience and Biobehavioral Reviews*.

[2] Scheer FAJL, Buijs RM (1999). Light affects morning salivary cortisol in humans. *Journal of Clinical Endocrinology and Metabolism*.

[3] Ishida A, Mutoh T, Ueyama T (2005). Light activates the adrenal gland: timing of gene expression and glucocorticoid release. *Cell Metabolism*.

[4] Dickmeis T (2009). Glucocorticoids and the circadian clock. *Journal of Endocrinology*.

[5] Burgess HJ, Eastman CI (2005). The dim light melatonin onset following fixed and free sleep schedules. *Journal of Sleep Research*.

[6] Burgess HJ, Savic N, Sletten T, Roach G, Gilbert SS, Dawson D (2003). The relationship between the dim light melatonin onset and sleep on a regular schedule in young healthy adults. *Behavioral Sleep Medicine*.

[7] Crowley SJ, Acebo C, Fallone G, Carskadon MA (2006). Estimating dim light melatonin onset (DLMO) phase in adolescents using summer or school-year sleep/wake schedules. *Sleep*.

[8] Wilhelm I, Born J, Kudielka BM, Schlotz W, Wüst S (2007). Is the cortisol awakening rise a response to awakening?. *Psychoneuroendocrinology*.

[9] Sharkey KM, Carskadon MA, Figueiro MG, Zhu Y, Rea MS (2011). Effects of an advanced sleep schedule and morning short wavelength light exposure on circadian phase in young adults with late sleep schedules. *Sleep Medicine*.

[10] Bierman A, Klein TR, Rea MS (2005). The Daysimeter: a device for measuring optical radiation as a stimulus for the human circadian system. *Measurement Science and Technology*.

[11] Portaluppi F, Touitou Y, Smolensky MH (2008). Ethical and methodological standards for laboratory and medical biological rhythm research. *Chronobiology International*.

[12] Pruessner JC, Kirschbaum C, Meinlschmid G, Hellhammer DH (2003). Two formulas for computation of the area under the curve represent measures of total hormone concentration versus time-dependent change. *Psychoneuroendocrinology*.

